# The Ethiopian Surgical Outcome Study (Ethio-SOS): a 7-day multicentre national prospective observational cohort study

**DOI:** 10.1136/bmjgh-2025-020147

**Published:** 2025-09-29

**Authors:** Atalel Fentahun Awedew, Fitsum Kifle Belachew, Katherine R Iverson, Tesfay Yohannes Ambese, Kokeb Desita Belihu, Abiy Dawit Tantu, Leake Gebrargs Gebreslase, Masresha G Teklehaimanot, Kalkidan Kifle, Nigat Amsalu Addis, Peniel Kenna Dula, Bruce Biccard, Andualem Deneke, On behalf of the Ethiopian Surgical Outcomes Study (Ethio-SOS) investigators, Atalel Fentahun Awedew

**Affiliations:** 1 Surgery, Debre Tabor University, Debre Tabor, Ethiopia; 2 Network for Perioperative and Critical Care, Asrat Woldeyes Health Sciences Campus, Debre Berhan University, Debre Birhan, Ethiopia; 3 Surgery, Medical College of Wisconsin, Milwaukee, Wisconsin, USA; 4 Department of Statistical and Data Governance, Global Partners for Improving Surgical System (GPISS), Project Network for Perioperative and Critical Care (GPISS-N4PCc), Addis Ababa, Ethiopia; 5 Anesthesia, Debre Berhan University, Debre Birhan, Amhara, Ethiopia; 6 Ethiopia Ministry of Health, Addis Ababa, Addis Ababa, Ethiopia; 7 Anesthesia, Axum University, Axum, Ethiopia; 8 Department of Anesthesia, Mekelle University, Mekelle, Ethiopia; 9 Statistical and Data Governance, Global Partners for Improving Surgical System (GPISS), Network for Perioperative and Critical Care (GPISS-N4PCc), Addis Ababa, Ethiopia; 10 Gynecology and Obstetrics, University of Gondar College of Medicine and Health Sciences, Gondar, Amhara, Ethiopia; 11 Clinical Research and Clinical Trial Management, Global Partners for Improving Surgical System (GPISS), Network for Perioperative and Critical Care (GPISS-N4PCc), Addis Ababa, Ethiopia; 12 Department of Anaesthesia and Perioperative Medicine, University of Cape Town Faculty of Health Sciences, Observatory, Western Cape, South Africa; 13 Department of Surgery, School of Medicine, College of Health Sciences, Addis Ababa University, Addis Ababa, Oromia, Ethiopia; 14 Surgery, Debre Tabor University, Debre Tabor, Amhara, Ethiopia

**Keywords:** Global Health, Surgery

## Abstract

**Introduction:**

Safe surgical care is a cost-effective intervention for addressing a wide range of conditions, yet postoperative complications remain a leading global cause of disability, mortality and economic loss, disproportionately affecting low- and middle-income countries. This study aims to generate robust epidemiological data on postoperative outcomes for surgical patients in Ethiopia.

**Method:**

This 7 day national observational cohort study included adult patients undergoing elective and non-elective surgeries, using a convenience sampling method to recruit as many hospital sites as possible from all regions of Ethiopia. The primary outcomes measured were 7 day in-hospital mortality and postoperative complications. Statistical analysis included descriptive statistics and logistic regression models to identify risk factors for mortality and complications.

**Results:**

A total of 4412 surgical patients across 46 Ethiopian hospitals were included in this study. The median patient age was 30 years (IQR: 25–42), with a predominance of female participants 2772/4412 (62.8%) and American Society of Anaesthesiologists (ASA) classification class I–II classifications. The overall complication rate was 19.8% (873/4412), with 4.2% (184/4412) experiencing severe complications (Clavien-Dindo grades III–IV) necessitating reoperation. The overall mortality rate was 0.4% (17/4412), with a median age at death of 40 years (IQR=29–49). Our findings suggest that the key drivers of perioperative mortality and postoperative complications were higher ASA class, comorbidities, cancer surgery, infections and emergency surgical procedures.

**Conclusion:**

One in five surgical patients in Ethiopia experiences postoperative complications and a high rate of reoperation, despite exhibiting a low-risk profile, young age and a low rate of high-risk surgical procedures. This suggests a need for more evidence-based interventions to strengthen the foundations, care processes and quality of the surgical system to achieve safe and effective care and improve overall surgical outcomes in the country.

**WHAT IS ALREADY KNOWN ON THIS TOPIC**

- Following landmark movements of the Lancet Commission on Global Surgery report in 2015, and the WHO General Assembly resolution WHA68.15 initiatives, the government of Ethiopia has demonstrated a clear commitment to improving surgical care by adopting surgical health as a national public health priority. This commitment is evidenced by the development and implementation of the Saving Lives Through Safe Surgery (SaLTS) programme (2016–2020) and its successor, SaLTS II (2021–2025). To assess the existing state of surgical healthcare in Ethiopia and to contextualise our current research, we conducted a thorough literature review. While our search revealed the existence of broader surgical outcome studies like the International and African Surgical Outcomes Studies (ISOS and ASOS), we found a notable scarcity of data specifically addressing surgical outcomes within Ethiopia. Though ASOS provides some insights with limited Ethiopian participation, the overall evidence base remains fragmented and insufficient to provide a comprehensive understanding of the country’s surgical landscape. Recognising that increasing access to surgery is a national priority for Ethiopia, it is equally crucial to ensure that surgical procedures are safe and effective, thereby preventing unnecessary perioperative morbidity and mortality. The relative dearth of comprehensive surgical outcomes data across all regions of Ethiopia highlights the urgent need for a robust epidemiological study focused on perioperative patient outcomes following surgery.

**WHAT THIS STUDY ADDS**

- The Ethiopian Surgical Outcomes Study gathered comprehensive data from 46 hospitals across the country, focusing on all in-patient surgical procedures. The study found that one in five surgical patients in Ethiopia experiences postoperative complications and 21% of these patients with complications required reoperations. Interestingly, despite the demographic profile of these patients being predominantly younger and generally lower risk, the incidence of complications remained notably high. Furthermore, our findings indicated that, in comparison to surgical outcomes reported in several other African countries, as well as on a continental and global scale, the mortality rate for patients undergoing surgery in Ethiopia was significantly lower. This suggests that, while complications are prevalent, the overall survival rates postsurgery are more favourable than one might expect given the context. These results underscore the need for a deeper understanding of the factors contributing to these outcomes, including potential differences in surgical practices, postoperative care and patient management strategies. Additionally, they highlight the importance of continued efforts to improve surgical quality and safety in Ethiopia, ensuring that advancements in surgical access are matched by enhancements in patient care and outcomes.

**HOW THIS STUDY MIGHT AFFECT RESEARCH, PRACTICE OR POLICY**

- The Ethiopia Surgical Outcomes Study has shed light on the complex landscape of surgical outcomes in Ethiopia, revealing critical insights into patient care and postoperative results. Our research indicates a concerning prevalence of potentially avoidable postoperative complications, particularly among low-risk patients. These complications appear to stem largely from an inadequate implementation of evidence-based strategies for complication prevention during the perioperative period. A significant contributing factor to this issue is the limited availability of both human and hospital resources, which hampers the ability to provide optimal care. While the global safe surgery campaign has positively influenced surgical practices and our findings show a relatively low mortality rate, the data also suggest that the incidence of postoperative complications remains unacceptably high. This situation underscores the urgent need to address the perioperative care of patients, especially those with deteriorating physiological conditions. To improve surgical outcomes in Ethiopia, it is essential to develop and implement a nationwide surgical quality improvement strategy. Such an initiative could focus on enhancing perioperative care, ensuring that sufficient resources are allocated to support healthcare providers in delivering high-quality care. By prioritising these efforts, we could significantly reduce postoperative complications and ultimately save many lives following surgery in Ethiopia.

## Introduction

 Surgical conditions are a significant contributor to global mortality and morbidity, particularly in low- and middle-income countries (LMICs).^1^ Given that surgically treatable conditions account for 28%–32% of the global disease burden,^1^ safe and effective surgery is crucial to address this health priority. However, postoperative complications remain a major cause of disability, death and economic loss, disproportionately affecting LMICs.^2 3^ Globally, surgery-related deaths exceed 4.2 million annually, with over half occurring in LMICs.^2^ The WHO resolution WHA 68.15^4 5^ and the Lancet Commission on Global Surgery (LCoGS)^1^ have significantly advanced surgical health through advocacy, quality indicator development, policy formulation and capacity building in LMICs since their advent in 2015. Recognising the high burden of surgical conditions and aligned with the global surgical movement, the Ethiopian government has prioritised surgical care within its broader healthcare strategy. This commitment is demonstrated through the implementation of the National Surgical, Obstetric and Anaesthesia Plan (NSOAP) framework, initially through the Saving Lives Through Safe Surgery (SaLTS I) programme (2016–2020), aiming to strengthen surgical systems in Ethiopia. Building on this foundation, SaLTS II (2021–2025) aims to further expand surgical capacity, improve quality and enhance accessibility.^6 7^

Despite the global surgery movement gaining prominence, perioperative surgical outcomes remain understudied globally and particularly in Africa. Some studies, including the African Surgical Outcomes Study (ASOS)^8^ and the International Surgical Outcomes Study (ISOS),^9^ have provided valuable insights into perioperative outcomes and mortality rates in different healthcare settings. The ASOS found that African surgical patients experience a disproportionately high perioperative mortality rate despite a lower burden of comorbidities compared with high-income countries (HICs). However, perioperative outcome data specific to Ethiopia remain limited and there was under-representation of Ethiopian data in these broader regional studies. Therefore, the national perioperative epidemiological data on patient characteristics and surgical outcomes still warrant closer investigation.^10 11^ There is also a need to understand the impact of Ethiopia’s strategic surgical care programmes, including SaLTS I and SaLTS II, on improving surgical outcomes and quality of care. To address these gaps, we conducted the first large-scale national surgical outcome evaluation in Ethiopia, aiming to generate epidemiological data on the incidence of postoperative complications among surgery patients. The findings will inform future research and healthcare policies, enabling policymakers to target interventions, allocate resources more effectively, improve surgical care quality and progress towards achieving Universal Health Coverage.

## Methods

### Study design, setting, and participants

From January to April 2024, a 7 day, national, prospective, observational cohort study was conducted, involving all patients undergoing various types of inpatient surgery across all regions of Ethiopia. This study was conducted in public hospitals across Ethiopia that provide surgical services, including tertiary, general and primary hospitals. Local investigators, including surgeons, anaesthetists, obstetricians, SaLTS leaders, general practitioners and nurses, managed data procedures, overseeing participant recruitment and ensuring informed consent and timely data collection. All patients undergoing elective and non-elective surgery with a planned overnight hospital stay following surgery during the study week were eligible for inclusion. Patients undergoing planned day surgery, paediatrics (age<18 years) or radiological procedures not requiring anaesthesia were excluded. The 7 day post-operative data collection period was chosen for consistency with the ASOS.^8^ This study complies with the STrengthening the Reporting of OBservational studies in Epidemiology (STROBE) statement^12^ (online supplemental Appendix 1).

### Variables and data

To facilitate a direct comparison of surgical outcomes data between Ethiopia and broader African studies^13^ and the global landscape,^9^ the study design mirrored that of a previous international study, ensuring compatibility across patient data sets including common indications for surgery and procedure types. Surgical complications were rigorously assessed using predefined criteria and graded according to severity, utilising both a mild, moderate and severe scale,^14^ as well as the established Clavien-Dindo classification system.^15^ The specific definitions of all data sets and variables employed in this study are detailed in the online supplemental Appendix 2.

### Data collection tool and procedures

The data collection tool was designed based on previous ISOS,^8 9 16^ with minor modifications to ensure relevance to the Ethiopian surgical setting. It captured key variables across the preoperative, intraoperative and postoperative phases of surgical care. Before the study commenced, all investigators participated in a standardised virtual training programme organised by the National Perioperative Quality Improvement Network. The training covered several critical topics, including study objectives, methodology and ethical considerations; proper use of paper and electronic case record forms (CRFs); standardised definitions for key postoperative outcomes; data verification and quality control procedures and effective use of the Research Electronic Data Capture (REDCap) system for electronic data entry and management. Data collection at each hospital was conducted using paper CRFs, which were subsequently transcribed onto the REDCap system. During electronic data entry, each hospital was assigned a numeric code, and each patient received a unique identifier code, enabling local investigators to track specific patients while maintaining confidentiality. On auditing data and cross-checking when missingness was observed, any discrepancies were resolved through communication with the site lead investigators to ensure data accuracy and completeness.

### Outcomes

The outcome measures included seventh day in-hospital mortality, postoperative complications and prevalence of critical illness. Detailed tracking of complications severity, such as infectious complications and cardiovascular complication, was assessed.

### Statistical analysis

There was no prespecified sample size and the study used a convenience sampling technique to recruit as many inpatient surgery patients as possible from various hospitals across Ethiopia.

Data are presented with graphs and tables. Categorical variables are summarised as proportions, using χ^2^ or Fisher’s exact tests for comparisons. Continuous variables are described by distribution: normally distributed as means with SD; non-normally distributed as medians with IQRs. Univariate analysis identified factors linked to postoperative complications and in-hospital mortality. A multivariable logistic regression model determined factors independently associated with these outcomes while controlling for confounders. Factors included were based on significant associations in univariate analysis. Logistic regression results are shown as adjusted OR with 95% CIs. Mixed-effects logistic regression with random hospital intercepts was initially applied to address potential hospital-level clustering. Hospital-level variability was quantified via the intraclass correlation coefficient, while model fit used likelihood ratio tests and compared standard logistic regression using Akaike and Bayesian information criteria. Due to minimal hospital-level clustering contribution, standard logistic regression was used for further analyses. The validation cohort was evaluated by calculating the area under the receiver operating characteristic (ROC) curve to assess discrimination. A generally accepted approach suggests that an area under the ROC curve or C statistic of less than 0.60 reflects poor discrimination; 0.60–0.75, possibly helpful discrimination and more than 0.75, clearly useful discrimination.^17^ To assess calibration, the Hosmer–Lemeshow test was utilised; a non-significant result from this test signifies good calibration of the model. Data analysis was conducted using Stata V.17.

### Role of the funding source

The funder of the study had no role in study design, data collection, analysis, interpretation or the writing of the report

## Results

**Figure 1:**
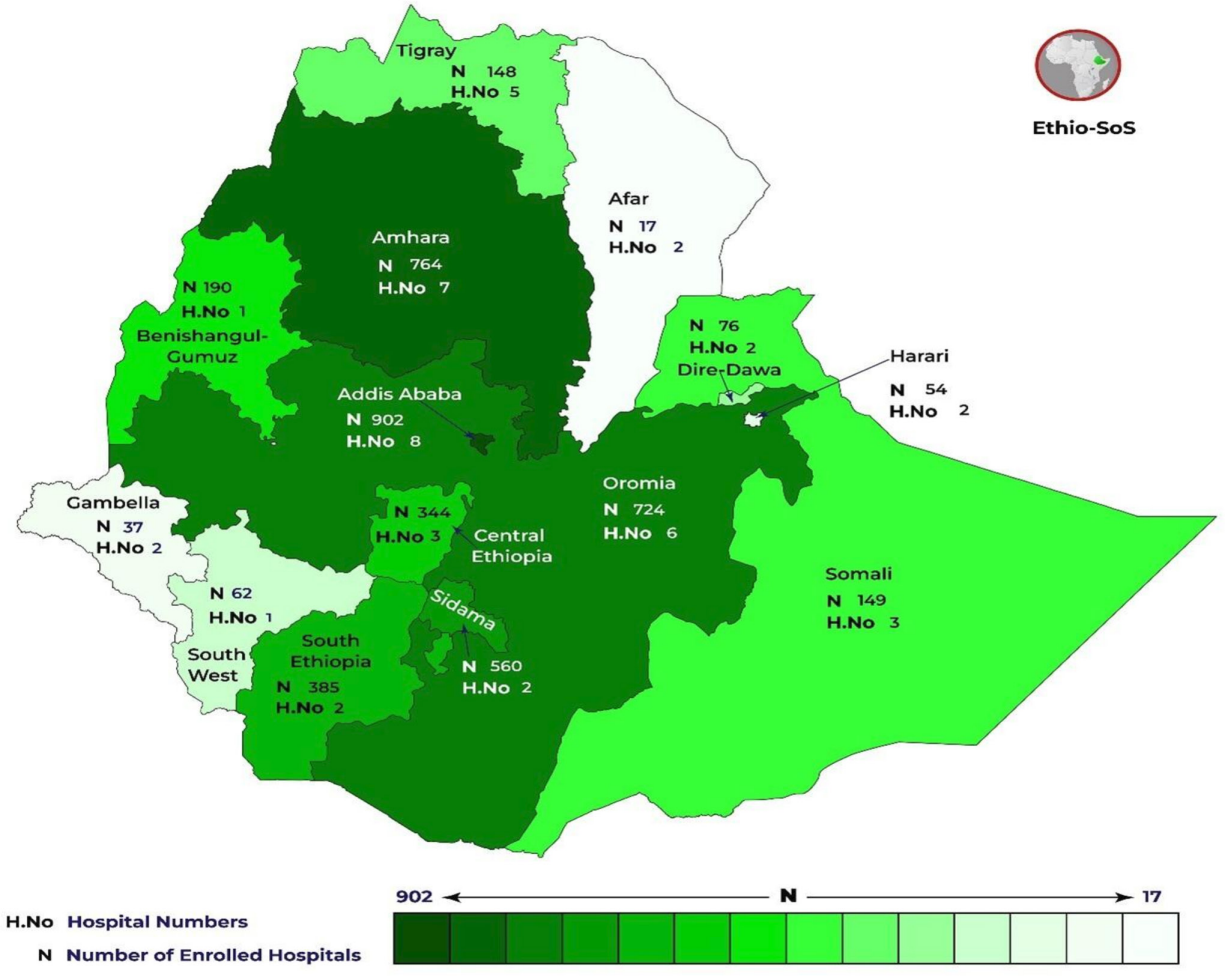
Participating Hospitals in the Ethiopian Surgical Outcomes Study.

We recruited 4412 patients from 46 hospitals participated hospitals, comprising 22 specialised hospitals, 21 general hospitals and three primary hospitals across various regions of Ethiopia. The number of facilities from each region is illustrated in figure 1, online supplemental Figure S1 and Table S8.

The median age of the participants was 30 years (IQR=25–42), with the majority 2772/4412 (62.8%) identifying as female. Most participants were classified under the American Society of Anesthesiologists (ASA) classification as class I 2114/4412 (47.9%) and class II 2074/4412 (47.0%). Regarding the types of surgeries performed (56%) 2470/4412 were classified as major procedures, followed by intermediate surgeries (37.2%, 1638/4412). Almost half of the surgeries were emergent surgery cases (47%, 2073/4412), while (31%) 1361/4412 were elective surgery (table 1).

**Table 1:** ·Baseline characteristics of the Ethiopia Surgical Outcomes Study patient cohort

Baseline variable	All patients (n=4412)	Patients with complications (n=873)	Patients with no complications (n=3538)	Patients who died (n=17)	Patients who survived (n=4395)
Age (median±IQR)	30 (25–42)	32 (25–46)	30 (25–40)	40 (29–49)	30 (25–42)
Age category					
18–29	2085 (47.3%)	349 (40.0%)	1736 (49.1%)	5 (29.4%)	2080 (47.3%)
30–69	2142 (48.5%)	468 (53.6%)	1673 (47.3%)	9 (52.9%)	2133 (48.5%)
≥ 70	185 (4.2%)	56 (6.4%)	129 (3.6%)	3 (17.6%)	182 (4.1%)
**Sex**					
Male	1640 (37.2%)	400 (45.8 %)	1240 (35.1%)	9 (52.9%)	1631 (37.1%)
Female	2772 (62.8%)	473 (54.2%)	2298 (64.9%)	8 (47.1%)	2764 (62.9%)
**Current smoker**					
Yes	102 (2.3%)	45 (5.2%)	57 (1.6%)	0 (0%)	102 (2.3)
No	4304 (97.7%)	826 (94.8%)	3477 (98.4%)	17 (100%)	4287 (97.7%)
**Alcohol drinker**					
Yes	262 (6.0%)	99 (11.5%)	163 (4.7%)	2 (11.8%)	260 (6.0%)
No	4089 (94.0%)	764 (88.5%)	3324 (95.3%)	15 (88.2%)	4074 (94.0%)
**ASA score**					
I	2113 (47.9%)	425 (48.7%)	1688 (47.7%)	5 (29.4%)	2108 (48.0%)
II	2074 (47.0%)	370 (42.4%)	1704 (48.1%)	5 (29.4%)	2069 (47.1%)
III	176 (4.0%)	51 (5.8%)	124 (3.5%)	5 (29.4%)	171 (3.9%)
IV	26 (0.6%)	13 (1.5%)	13 (0.4%)	0 (0%)	26 (0.7%)
V	23 (0.5%)	14 (1.6%)	9 (0.3%)	2 (11.8%)	21 (0.5%)
**Severity of surgery**					
Minor	303 (6.9%)	41 (4.7%)	262 (7.4%)	0 (0%)	303 (6.9%)
Intermediate	1638 (37.2%)	249 (28.6%)	1389 (39.3%)	1 (5.9%)	1637 (37.3%)
Major	2470 (56.0%)	583 (66.7%)	1887 (53.3%)	16 (94.1%)	2454 (55.8%)
**Urgency of surgery**					
Elective	1363 (31.0%)	253 (29.0%)	1110 (31.4%)	3 (17.6%)	1360 (31.0%)
Urgent	971 (22.0%)	216 (24.8%)	755 (21.4%)	2 (11.8%)	969 (22.1%)
Emergency	2073 (47.0%)	403 (46.2%)	1670 (47.2%)	12 (70.6%)	2061 (46.9%)

Data are presented as n/N (%); age: median (IQR).

ASA, American Society of Anesthesiologists; CHF, congestive heart failure; CKD, chronic kidney disease; COPD, chronic obstructive pulmonary disease; IHD, ischaemic heart disease; PUD, peptic ulcer disease; PVD, peripheral vascular disease.

Common comorbidities on admission among the participants included hypertension (5.5%, 243/4412), diabetes mellitus (2.4%, 106/4412), solid tumours (1.1%, 48/4412) and HIV (1.1%, 47/4412). Obstetric surgeries were the most frequently performed (37.6%, 1661/4412), followed by orthopaedic surgeries (11.8%, 519/4412) and colorectal surgeries (11.3%, 498/4412). The WHO Surgical Safety Checklist was utilised in 78.0% (3438/4412) of the surgeries. The demographic characteristics of the participants are further detailed in online supplemental Table S1 and table 2.

**Table 2:** Surgical procedure category and postoperative outcomes in the Ethiopia Surgical Outcomes Study

Surgical procedure category					
**Surgical category**	**All patients** (**n=4412**)	**Patients with complications (n=873**)	**Patients with no complications (n=3538**)	**Patients who died (n=17**)	**Patients who survived (n=4395**)
Cardiac	2 (0.1%)	0 (0%)	2 (0.1%)	0 (0%)	2 (0.1%)
Colorectal	498 (11.3%)	129 (14.8%)	369 (10.4%)	4 (23.5%)	494 (11.2%)
ENT and maxillofacial	99 (2.2%)	33 (3.8%)	66 (1.9%)	0 (0%)	99 (2.2%)
Endocrine	204 (4.6%)	30 (3.4%)	174 (4.9%)	0 (0%)	204 (4.6%)
Gynaecology	201 (4.6%)	33 (3.8%)	168 (4.7%)	0 (0%)	201 (4.6%)
Hepatobiliary	194 (4.4%)	44 (5.0%)	150 (4.2%)	3 (17.6%)	191 (4.3%)
Neurosurgery	140 (3.2%)	27 (3.1%)	113 (3.2%)	0 (0%)	140 (3.2%)
Obstetrics	1661 (37.6%)	219 (25.1%)	1442 (40.8%)	3 (17.6%)	1658 (37.7%)
Ophthalmology surgery	66 (1.5%)	14 (1.6%)	52 (1.5%)	0 (0%)	66 (1.5%)
Orthopaedics	519 (11.8%)	132 (15.1%)	387 (10.9%)	1 (5.9%)	518 (11.8%)
Plastic surgery	71 (1.6%)	22 (2.5%)	49 (1.4%)	0 (0%)	71 (1.6%)
Thoracic	55 (1.2%)	13 (1.5%)	42 (1.2%)	0 (0%)	55 (1.2%)
UGI	243 (5.5%)	74 (8.5%)	169 (4.8%)	4 (23.5%)	239 (5.4%)
Urological surgery	290 (6.6%)	66 (7.6%)	224 (6.3%)	1 (5.9%)	289 (6.6%)
Vascular	46 (1.0%)	12 (1.4%)	34 (1.0%)	1 (5.9%)	45 (1.0%)
Other	121 (2.7%)	25 (2.9%)	96 (2.7%)	0 (0%)	121 (2.7%)
Surgical checklist	3438 (78.0%)	725 (83.1%)	2713 (76.8%)	14 (82.4%)	3424 (78.0%)

*Postoperative outcomes*					
	*Number of patients*	*Patients admitted to critical care immediately after surgery*	*Patients not admitted to critical care immediately after surgery*		
*All surgeries*					
Complications	873/4412 (19.8%)	137/652 (20.0%)	736/3756 (19.6%)		
Mortality	17/4412 (0.4%)	9/652 (1.4%)	8/3756 (0.2%)		
Critical care admission to treat complications	153/873 (17.5%)	114/153 (74.5%)	39/720 (5.4%)		
Death following a postoperative complication	13/873 (1.5%)	9/153 (5.9%)	4/720 (0.6%)		
**Elective surgeries only**					
Complications	253/1363 (18.6%)	32/156 (20.5%)	221/1205 (18.3%)		
Mortality	3/1363 (0.2%)	1/156 (0.6%)	2/1205 (0.2%)		
Critical care admission to treat complications	22/253 (8.7%)	16/22 (72.7%)	6/231 (2.6%)		
Death following a postoperative complication	2/253 (0.8%)	1/156 (0.6%)	1/97 (1.0%)		

ICU admission	Overall ICU admission	ICU admission immediately after surgery	ICU admission for postoperative complication management		
	153/4373 (3.5%)	72/4373 (1.6%)	81/4373 (1.8%)		
**ICU mortality**	10/153 (6.5%)	9/153 (5.8%)	1/153 (0.7%)		

Data presented as n/N (%).

ENT, ear, nose and throat; ICU, intensive care unit; UGI, upper gastrointestine.

### Postoperative outcomes

The overall postoperative complication rate was 19.8% (873/4412). Of these, 13/873 patients (1.5%) died. Approximately 153/873 patients (17.5%) with complications required admission to critical care, with about 114/153 (74.5%) being admitted to the ICU immediately after surgery, as shown in table 2. The presence of complications was associated with a prolonged hospital stay (median 3 days (IQR 2–4) without complications vs 4 days (3–6) with complications; p<0.0001). The most common postoperative complications were surgical site infections (SSIs), which were reported in 522/4408 cases (11.8%). This was followed by deep SSIs, with 216/4405 cases (4.9%), and postoperative bleeding, occurring in 126/4405 cases (2.9%). In terms of mortality by type of complications, infectious complications accounted for 6/698 (0.9%), cardiovascular complications resulted in 3/76 (3.9%) and other complications led to 8/269 (3.0%), as outlined in table 3.

**Table 3:** Postoperative complications in the Ethiopia Surgical Outcomes Study

Complications	Numbers of patients	Complication severity			Number of deaths for all patients who developed complications	Number of deaths for patients after elective surgery who developed complications
		Mild	Moderate	Severe		
**Infections**						
Superficial surgical site	4408	245 (5.6%)	229 (5.2%)	48 (1.1%)	2/522 (0.4%)	1/146 (0.7%)
Deep surgical site	4405	106 (2.4%)	72 (1.6%)	38 (0.9%)	1/216 (0.5%)	0/55 (0%)
Body cavity	4404	33 (0.7%)	46 (1.0%)	16 (0.4%)	1/95 (1.1%)	0/25 (0%)
Pneumonia	4406	31 (0.7%)	31 (0.7%)	16 (0.4%)	4/78 (5.1%)	1/22 (4.5%)
UTI	4406	58 (1.3%)	27 (0.6%)	6 (0.1%)	0/91 (0%)	0/41 (0%)
Bloodstream	4406	48 (1.1%)	28 (0.6%)	17 (0.4%)	4/93 (4.3%)	1/30 (3.3%)
Total					**6/698 (0.9%)**	**2/200 (1.0%)**
**Cardiovascular complications**						
Myocardial infarction	4406	5 (0.1%)	7 (0.2%)	2 (0.05%)	0/14 (0%)	0/6 (0%)
Arrhythmia	4407	5 (0.1%)	8 (0.2%)	7 (0.2%)	1/20 (5.0%)	0/5 (0%)
Pulmonary oedema	4406	7 (0.2%)	4 (0.1%)	6 (0.14%)	1/17 (5.9%)	0/8 (0%)
Pulmonary embolism	4406	7 (0.16%)	10 (0.2%)	8 (0.18)	0/25 (0%)	0/8 (0%)
Stroke	4408	7 (0.16%)	4 (0.1%)	4 (0.1%)	0/15 (0%)	0/3 (0%)
Cardiac arrest	4206	NA	NA	22 (100%)	2/22 (8.69%)	0/7 (0%)
Total					**3/76 (3.9%)**	**0/22 (0%)**
**Other complications**						
GI bleeding	4407	12 (0.3%)	3 (0.1%)	6 (0.14%)	1/21 (4.8%)	0/5 (0%)
Acute kidney injury	4407	32 (0.7%)	10 (0.2%)	8 (0.18%)	4/50 (8.0%)	1/16 (6.3%)
Postoperative bleeding	4405	68 (1.5%)	38 (0.9%)	12 (0.3%)	1/118 (0.8%)	0/42 (0%)
ARDS	4407	11 (0.3%)	7 (0.2%)	7 (0.2)	2/25 (8.0%)	0/9 (0%)
Anastomotic leak	4407	15 (0.3%)	8 (0.18%)	10 (0.23)	1/33 (3.0%)	0/11 (0%)
All others	4224	32 (0.8%)	27 (0.6%)	18 (0.4%)	2/77 (2.6%)	0/22 (0%)
Total					**8/269 (3.0%)**	**1/86 (1.2%)**

Data are presented as n/N (%).

ARDS, acute respiratory distress syndrome; GI, gastrointestinal; NA, not applicable; UTI, urinary tract infections.

Non-communicable diseases were the most common indication for surgery, accounting for 1724 (39.1%). Infectious indications for surgery were associated with significantly higher complication rates, whereas caesarean sections demonstrated comparatively fewer complications (online supplemental Table S2). The comparison between our findings and those from the ASOS, Nigerian Surgical Outcome Study (NiSOS) and ISOS cohorts is presented in online supplemental Table S3. Severe complications (grades III–IV) requiring reoperation occurred in 4.2% of patients (184/4412) from all surgical patients. Most complications, however, were grade I, with 282/873 (32.4%) and 406/873 (46.5%) grade II (online supplemental Table S4).

Additionally, 3.5% of patients (153/4373) were admitted to the ICU due to critical illness, with 1.6% (72/4373) being admitted immediately after surgery and 1.8% (81/4373) requiring ICU care for the management of postoperative complications, as detailed in table 2.

**Figure 2:**
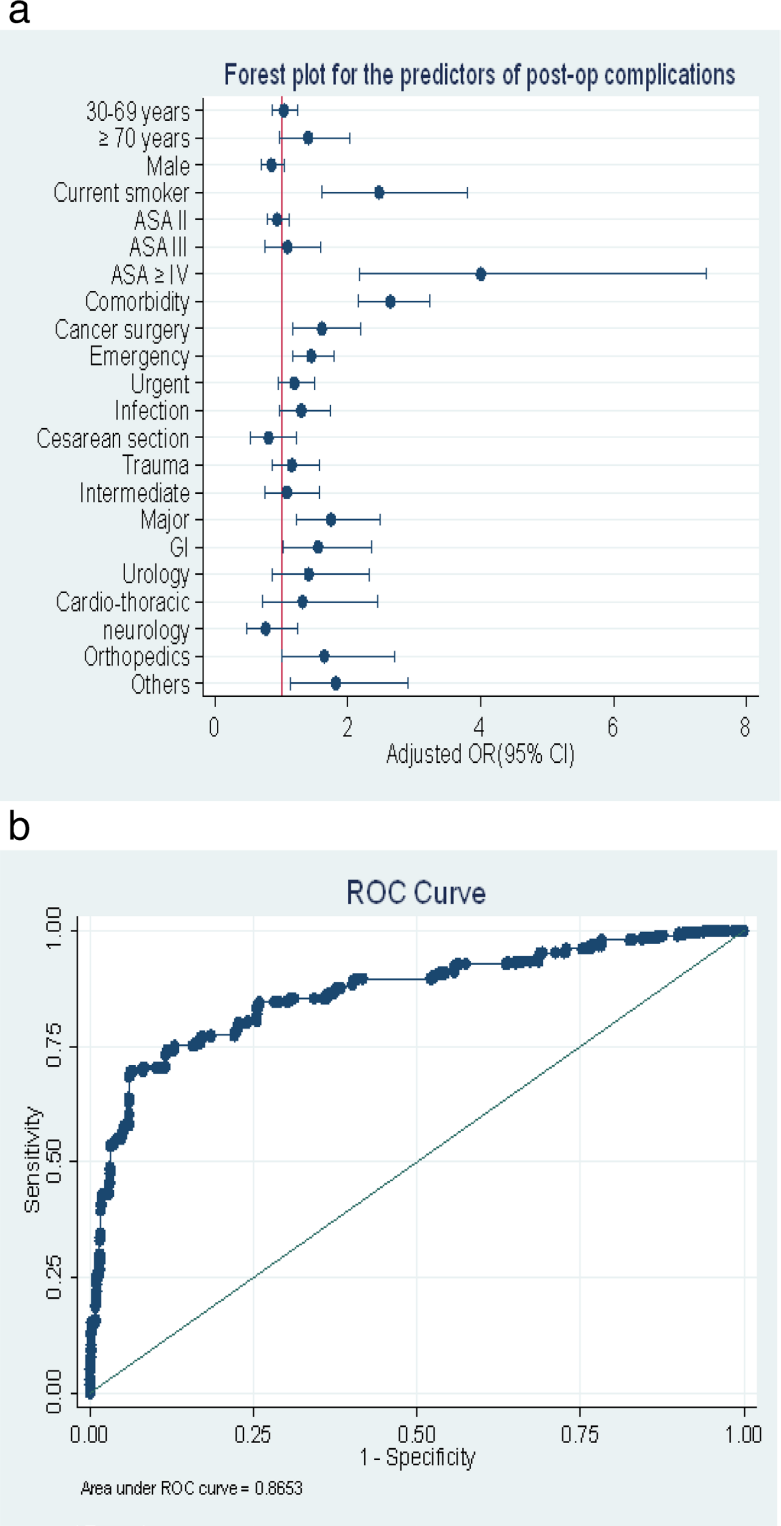
Model of the study (a) Forest plot of predictors of postoperative complications, (b) Area under the ROC for EthioSOS calculator).

Multivariable logistic regression analysis identified several independent predictors of postoperative complications presented at figure 2, online supplemental Table S5.

ASA scores≥IV demonstrated the strongest association with complications (AOR=4.0, 95% CI 2.17 to 7.40; p<0.001), followed by the presence of comorbidities (AOR=2.63, 95% CI 2.15 to 3.23; p<0.001) and current smoking status (AOR=2.47, 95% CI 1.60 to 3.80; p<0.001). Surgical urgency significantly influenced outcomes, with emergency procedures associated with higher complication odds compared with elective surgeries (AOR=1.44, 95% CI 1.16 to 1.79; p=0.001). Major surgeries (AOR=1.74, 95% CI 1.38 to 1.92; p=0.002) and cancer-related procedures (AOR=1.60, 95% CI 1.17 to 2.19; p=0.003) also independently predicted complications.

### Mortality

Among the 4412 surgical patients, the overall mortality rate was 0.4% (17/4412), with a median age of 40 years (IQR=29–49) at the time of death. Mortality rates varied significantly depending on the type of surgery as detailed in table 1. Emergency surgeries had a higher mortality rate of 0.6% (12/2073), compared with 0.2% (3/1363) for elective surgeries (table 1). ICU admission was associated with a notably higher mortality rate of 6.5% (10/153) (table 2).

Mortality was also stratified based on patient characteristics. Patients classified as ASA I and II had a mortality rate of 0.24% (10/4187), while those with an ASA score of III or higher had a significantly increased mortality rate of 3.11% (7/225). Among patients who experienced postoperative complications, the mortality rate was 1.5% (13/873) versus 0.11% (4/3539) for those with no complications. Patients with comorbidities had a significantly higher mortality rate of 0.96% (6/628) than those without comorbid conditions (0.29%, 11/3764), with an OR of 3.29 (1.21,8.93) (online supplemental online supplemental Table S6). Postoperative complications and mortality of surgery across regions and hospital type are presented in online supplemental online supplemental Table S7 and S8.

## Discussion

The primary finding of this study reveals that surgical patients in Ethiopia are generally younger, possess a low risk profile and experience elevated complication rates, while maintaining low mortality rates. Approximately one in five surgical patients in Ethiopia experiences a postoperative complication within 7 days of surgery, and one in 67 of these patients died. Our findings indicate that the rate of postoperative complications following surgery in Ethiopia is slightly higher than that observed in the ISOS (16.8%)^9^ and similar to rates reported in other African studies.^8 13 18^ While limited data on postoperative outcomes exist for African nations,^8 13 18^ our interpretation aligns with findings from Nigeria (18.5%),^18^ South Africa^13^ and a multicountry study of 25 African nations (18.2%).^8^ These epidemiological studies reported high complication rates even though patients often presented with sociodemographic characteristics typically associated with good recovery, such as low-risk profiles, low lower risk procedures and younger age. This suggests that factors beyond individual patient physiological health are at play. As defined by the LCoGS, improving surgical quality requires attention to structures, processes and outcomes,^1^ and the Lancet Global Health Commission recommended to strength foundation, process of care and quality impact evaluation to effective, safe, accessible and affordable surgical care in LMICs to save millions of lives.^19^ Our results, combined with findings from previous African studies, provide important insights into specific processes and outcomes that warrant targeted attention in Ethiopia and other LMICs to provide effective, safe, accessible and affordable surgical care in LMICs, with the potential to save millions of lives.^19^ A significant proportion of complications in our study occurred in the days immediately following surgery, suggesting that many are potentially preventable. Despite the higher rate of overall complications, our results indicate that the mortality rate following surgery in Ethiopia was significantly lower than that observed in other African nations (Nigeria, Kenya and South Africa),^13 18 20^ as well as in ISOS^9^ and ASOS.^8^ The mortality rate observed in our study aligns with previous assessments of surgical outcomes in two specific regions of Ethiopia (Sidama and Amhara),^21^ which reported a similar rate of approximately 0.4%. This relatively low mortality rate is not necessarily indicative of a fully mature Ethiopian surgical system, but rather, as compared with the aforementioned studies,^8^ our surgical patient population has undergone fewer high-risk surgical procedures such as cardiac, vascular, oncological and other complex interventions. Drawing insights from our study and previous surgical outcome studies in LMICs, it is clear that while increasing access to surgery is crucial for addressing the significant global burden of surgical conditions, ensuring the safety and effectiveness of these surgical treatments is paramount.^22^

Intensive care unit (ICU) admission rates for surgical patients are a key indicator of surgical service quality. Our study found a 3.5% ICU admission rate, approximately half that of the South African Surgical Outcomes Study (6.5%)^13^ and the European Surgical Outcome Report (7.7%).^16^ Compared with the African Surgical Outcomes Study (2.8%) and the Nigerian Surgical Outcomes Study (2.4%), our rate was slightly higher. These differences likely stem from our cohort’s lower risk profile, fewer complex surgeries and limited ICU resources. Additionally, despite Ethiopia’s high WHO Surgical Safety Checklist adherence (78%) compared with ASOS (57.1%)^8^ and NiSOS (56.1%), adherence still falls below the standard. Given evidence that checklist adherence reduces postoperative mortality and complications, focused training, teamwork investment, enhanced communication and collective learning are crucial.^23^ Leadership engagement is also essential to foster a supportive environment for safe surgery implementation.^23^

Our findings also indicated that approximately 21% of patients experienced complications classified as Clavien-Dindo grade III or higher, requiring reoperation via radiological or open surgical intervention and, in some cases, ICU admission. This rate exceeds international benchmarks, as global surgical quality standards recommend that Clavien-Dindo grade II and above should remain below 8% following surgical procedure.^24^ Our results suggest that the key drivers of perioperative mortality and morbidity are largely consistent across Africa^8 13 18^ and international studies,^9 16^ which include higher ASA class, comorbidities, cancer surgery, infections and surgical timing. The increased risk of mortality related to patient comorbidities such as hypertension, cancer, diabetes and HIV is consistent with other studies in Ethiopia^25 26^ and well established globally. This underscores the need for national quality improvement programmes and the implementation of surgical-risk calculators to improve postoperative surveillance and proactively manage high-risk patients.

The findings of elevated complication rates likely stem in part from a lack of dedicated resources—including supplies, infrastructure, workforce and effective governance—necessary for establishing a foundation of quality surgical care.^19^ Given these complications are present in the initial 7 day postoperative period, this likely reflects limitations in staff and physician ability to ensure routine safety and quality due to these multifaceted resource restraints. Prior research highlights this deficiency: the median number of 0.7 surgeons, obstetricians and anaesthesiologists per 100 000 population in Africa falls significantly below the 20–40 specialists per 100 000 population deemed necessary to meaningfully reduce perioperative mortality, complications and surgical disabilities.^1 8^ To address these challenges, it is imperative to implement evidence-based and effective care strategies which create robust systems that prioritise safety, prevention and early detection, continuity and integration of care, timely intervention and population health management throughout the continuum of surgical care.^19^ This includes adopting interventions, such as early warning scores, critical-care outreach teams, medical emergency response teams and targeted critical-care skills training for junior surgeons, which have become standard practice in HICs and are increasingly being adapted in LMICs.^8^ The Ethiopian SaLTS 2025 surgical targets aimed to reduce complications—primarily SSIs to less than 5%. Therefore, this foundational study provides essential baseline data for revising Ethiopia’s surgical strategy, guiding future SaLTS updates and strengthening the essential foundations, core care processes and demonstrable quality impact within the country’s surgical system.^19^

Our study possesses certain limitations that warrant consideration. The 7 day cohort design restricts the window of observation, potentially underestimating the incidence of long-term postoperative mortality and morbidity. Clinical experience suggests that numerous infectious and nutritional complications often manifest beyond this 7 day period, impacting the overall burden of adverse outcomes. Beyond the short observation window, the study’s generalisability may be constrained by the relatively small number of high-risk surgical procedures such as cardiac and vascular surgery included in the sample. This could limit the applicability of the findings to more complex cases. Additionally, rates of SSI are reported without association of wound classification for each operation, which is predictive of postoperative infection rates. This limits the interpretation of infection data from this study but remains consistent with prior ASOS and ISOS data sets. In addition, the study’s data collection did not encompass critical outcomes such as the length of hospital and ICU stays. Our multivariate analysis is limited without the incorporation of a few potentially influential factors including level of facility or surgical provider; however, this reflects the skewed distribution of facilities and practitioners in this study and is consistent with ASOS and ISOS methodology. Furthermore, the ongoing conflict in many parts of the country has significantly impacted disease patterns, healthcare functionality, the care delivery system and the distribution and migration of the healthcare workforce, resulting in grave impact on the surgical outcomes.

The Ethiopian Surgical Outcomes Study (EthioSOS) provides valuable insights into the current state of surgical care in Ethiopia, highlighting areas requiring immediate attention and strategic interventions. Our study highlights a high rate of postoperative complications, coupled with elevated reoperation, ICU admission rates and underutilisation of the WHO Surgical Safety Checklist. Given that the majority of deaths and postoperative complications occur in the immediate postoperative period, and despite surgical patients in Ethiopia tending to be younger with lower overall risk profiles, these findings underscore the urgent need to enhance patient safety through improved postoperative surveillance for deteriorating patients on the ward. As the LCoGS^1^ advocates for expanded access to safe, accessible and affordable surgical care, our study findings indicate the critical importance of adopting and effectively implementing the Lancet Global Health Commission’s framework of essential foundations, robust care processes and demonstrable quality impact^19^ within the surgical care system to achieve this objective in Ethiopia.

## Supplementary material

10.1136/bmjgh-2025-020147 Supplementary file 1

10.1136/bmjgh-2025-020147 Supplementary file 2

10.1136/bmjgh-2025-020147 Supplementary file 3

10.1136/bmjgh-2025-020147 online supplemental file 4

## Data Availability

Data are available upon reasonable request.
