# Supplementary material for: The Ethiopian Surgical Outcome Study (Ethio-SOS): a 7-day multicentre national prospective observational cohort study
Source: BMJ Glob Health. 2025 Sep 29;10(9):e020147. doi: 10.1136/bmjgh-2025-020147 (PMC12481335; doi:10.1136/bmjgh-2025-020147)
Supplement: Supplementary file 2 [file bmjgh-10-9-s002.docx]

**Appendix 2: supplement method and results to ‘’The Ethiopian Surgical Outcome Study (Ethio-SOS): a 7-day Multicenter National Prospective Observational Cohort study ‘’**

Please note that portions of this supplement detailing **’The Ethiopian Surgical Outcome Study (Ethio-SOS): a 7-day Multicenter National Prospective Observational Cohort study’’** were copied from the supplementary content to the following publications

1. Biccard BM, Madiba TE, Kluyts HL, Munlemvo DM, Madzimbamuto FD, Basenero A, Gordon CS, Youssouf C, Rakotoarison SR, Gobin V, Samateh AL, Sani CM, Omigbodun AO, Amanor-Boadu SD, Tumukunde JT, Esterhuizen TM, Manach YL, Forget P, Elkhogia AM, Mehyaoui RM, Zoumeno E, Ndayisaba G, Ndasi H, Ndonga AKN, Ngumi ZWW, Patel UP, Ashebir DZ, Antwi-Kusi AAK, Mbwele B, Sama HD, Elfiky M, Fawzy MA, Pearse RM; African Surgical Outcomes Study (ASOS) investigators. Perioperative patient outcomes in the African Surgical Outcomes Study: a 7-day prospective observational cohort study. Lancet. 2018 Apr 21;391(10130):1589-1598. doi: 10.1016/S0140-6736(18)30001-1. Epub 2018 Jan 3. PMID: 29306587.
2. International Surgical Outcomes Study g. Global patient outcomes after elective surgery: prospective cohort study in 27 low-, middle- and high-income countries. British journal of anaesthesia. 2016;117(5):601-9.
3. Jammer I, Wickboldt N, Sander M, Smith A, Schultz MJ, Pelosi P, et al. Standards for definitions and use of outcome measures for clinical effectiveness research in perioperative medicine: European Perioperative Clinical Outcome (EPCO) definitions: a statement from the ESA-ESICM joint taskforce on perioperative outcome measures. European journal of anaesthesiology. 2015;32(2):88-105

# Appendix2: Method

**Hospital tier system in Ethiopia**

The Ethiopian healthcare system operates on a three-tiered structure, designed to provide increasing levels of specialized care to the population. At the base of the pyramid is the primary hospital, serving a catchment area of approximately 60,000 to 100,000 individuals. These facilities act as the first point of contact for most patients and are equipped to handle common medical conditions and basic surgical procedures. Moving up the hierarchy, the general hospital serves a significantly larger population base, ranging from 1 million to 1.5 million people. These hospitals offer a broader range of services and a higher level of specialization. At the apex of the system are tertiary or specialized hospitals, which provide the most advanced and complex medical care to a vast population of approximately 3.5 to 5 million individuals.

In terms of surgical capabilities, each tier is equipped to perform specific procedures aligned with its resources and expertise. Primary hospitals are typically capable of performing essential, " **Bellwether Procedures**" surgeries, including laparotomies for acute abdominal conditions, Cesarean sections for obstetric emergencies, and open bone debridement for managing infections. These procedures address common and life-threatening conditions at the community level. General hospitals possess a more extensive surgical portfolio, encompassing a wider range of specialties. They are typically equipped to perform general surgical procedures, obstetric and gynecological surgeries beyond basic C-sections, general orthopedic procedures to address fractures and dislocations, and basic traumatic brain surgeries to manage head injuries. These facilities represent a crucial step in providing more comprehensive surgical care within their respective regions. Finally, specialized hospitals are the hub for advanced surgical interventions, representing the pinnacle of surgical expertise within the country. These hospitals are capable of performing highly complex procedures, including hepatobiliary and pancreatic (HBP) surgeries for liver and pancreatic diseases, colorectal surgeries for managing complex bowel conditions, cardiothoracic surgeries for heart and lung diseases, advanced brain and spine surgeries for neurological conditions, and advanced orthopedic surgeries for complex bone and joint problems. This tiered system strives to ensure that patients receive the appropriate level of surgical care based on their specific needs, maximizing access and optimizing the utilization of resources across the country..

**Severity of Surgery**

- Minor surgery would include procedures lasting less than 30 minutes performed in a dedicated operating room which would often involve extremities or body surface or
  brief diagnostic and therapeutic procedures eg arthroscopy without intervention,
  removal of small cutaneous tumour, diagnostic proctology, biopsy of small lesions, etc.
- Intermediate procedures are more prolonged or complex that may pose the risk of
  significant complications or tissue injury. Examples include laparoscopic
  cholecystectomy, arthroscopy with intervention, bilateral varicose vein removal,
  tonsillectomy, inguinal hernia repair, breast lump resection, haemorrhoidectomy,
  appendicectomy, partial thyroidectomy, cataract surgery, uvuloplasty, minimally
  invasive repair of vaginal prolapse, vaginal hysterectomy, tendon repair of hand,
  fixation of mandibular fracture, etc.
- Major surgical procedures are expected to last more than 90 minutes and include major
  gut resection, major joint replacement, mastectomy, extensive head and neck tumour
  resection, abdominal aortic aneurysm repair, major vascular bypass procedure,
  procedures involving free flap to repair tissue defect, amputation, total thyroidectomy,
  cystectomy, trans-urethral resection of prostate, resection of liver tumour, carotid
  endarterectomy, nephrectomy, total abdominal hysterectomy, spinal discectomy, etc.

**Timing of Surgery**

Emergency – Need surgical intervention within 24 hours to save life or to prevent further complications with minimal investigations

Urgent -Need surgical intervention within 24+-72hours save life or to prevent further complications with adequate investigation and consultation

Elective -planned surgery after waiting weeks or months

Post operative complication grade with global accepted Clavien Dindo complication classification system

Grade I: Any deviation from the normal postoperative course without the need for pharmacological treatment or surgical, endoscopic, and radiological interventions Allowed therapeutic regimens are: drugs as antiemetics, antipyretics, analgetics, diuretics, electrolytes, and
physiotherapy. This grade also includes wound infections opened at the bedside
Grade II: Requiring pharmacological treatment with drugs other than such allowed for grade I complications. Blood transfusions and total parenteral nutrition are also included
Grade III : Requiring surgical, endoscopic or radiological intervention

- Grade IIIa: Intervention not under general anaesthesia
- Grade IIIb Intervention under general anaesthesia

Grade IV : Life-threatening complication (including CNS complications)* requiring IC/ICU management

- Grade IVa Single organ dysfunction (including dialysis)
- Grade IVb Multiorgan dysfunction

Grade V Death of a patient

***Dindo D, Demartines N, Clavien PA. Classification of surgical complications: a new proposal with evaluation in a cohort of 6336 patients and results of a survey. Ann Surg. 2004 Aug;240(2):205-13. doi: 10.1097/01.sla.0000133083.54934.ae. PMID: 15273542; PMCID: PMC1360123.***

***Current Definitions and ASA-Approved Examples***

| ***ASA PS Classification*** | ***Definition*** | ***Adult Examples, Including, but not Limited to:*** | ***Pediatric Examples, Including but not Limited to:*** | ***Obstetric Examples, Including but not Limited to:*** |
| --- | --- | --- | --- | --- |
| ***ASA I*** | ***A normal healthy patient*** | ***Healthy, non-smoking, no or minimal alcohol use*** | ***Healthy (no acute or chronic disease), normal BMI percentile for age*** |  |
| ***ASA II*** | ***A patient with mild systemic disease*** | ***Mild diseases only without substantive functional limitations. Current smoker, social alcohol drinker, pregnancy, obesity (30<BMI<40), well-controlled DM/HTN, mild lung disease*** | ***Asymptomatic congenital cardiac disease, well controlled dysrhythmias, asthma without exacerbation, well controlled epilepsy, non-insulin dependent diabetes mellitus, abnormal BMI percentile for age, mild/moderate OSA, oncologic state in remission, autism with mild limitations*** | ***Normal pregnancy*, well controlled gestational HTN, controlled preeclampsia without severe features, diet-controlled gestational DM.*** |
| ***ASA III*** | ***A patient with severe systemic disease*** | ***Substantive functional limitations; One or more moderate to severe diseases. Poorly controlled DM or HTN, COPD, morbid obesity (BMI ≥40), active hepatitis, alcohol dependence or abuse, implanted pacemaker, moderate reduction of ejection fraction, ESRD undergoing regularly scheduled dialysis, history (>3 months) of MI, CVA, TIA, or CAD/stents.*** | ***Uncorrected stable congenital cardiac abnormality, asthma with exacerbation, poorly controlled epilepsy, insulin dependent diabetes mellitus, morbid obesity, malnutrition, severe OSA, oncologic state, renal failure, muscular dystrophy, cystic fibrosis, history of organ transplantation, brain/spinal cord malformation, symptomatic hydrocephalus, premature infant PCA <60 weeks, autism with severe limitations, metabolic disease, difficult airway, long term parenteral nutrition. Full term infants <6 weeks of age.*** | ***Preeclampsia with severe features, gestational DM with complications or high insulin requirements, a thrombophilic disease requiring anticoagulation.*** |
| ***ASA IV*** | ***A patient with severe systemic disease that is a constant threat to life*** | ***Recent (<3 months) MI, CVA, TIA or CAD/stents, ongoing cardiac ischemia or severe valve dysfunction, severe reduction of ejection fraction, shock, sepsis, DIC, ARD or ESRD not undergoing regularly scheduled dialysis*** | ***Symptomatic congenital cardiac abnormality, congestive heart failure, active sequelae of prematurity, acute hypoxic-ischemic encephalopathy, shock, sepsis, disseminated intravascular coagulation, automatic implantable cardioverter-defibrillator, ventilator dependence, endocrinopathy, severe trauma, severe respiratory distress, advanced oncologic state.*** | ***Preeclampsia with severe features complicated by HELLP or other adverse event, peripartum cardiomyopathy with EF <40, uncorrected/decompensated heart disease, acquired or congenital.*** |
| ***ASA V*** | ***A moribund patient who is not expected to survive without the operation*** | ***Ruptured abdominal/thoracic aneurysm, massive trauma, intracranial bleed with mass effect, ischemic bowel in the face of significant cardiac pathology or multiple organ/system dysfunction*** | ***Massive trauma, intracranial hemorrhage with mass effect, patient requiring ECMO, respiratory failure or arrest, malignant hypertension, decompensated congestive heart failure, hepatic encephalopathy, ischemic bowel or multiple organ/system dysfunction.*** | ***Uterine rupture.*** |
| ***ASA VI*** | ***A declared brain-dead patient whose organs are being removed for donor purposes*** |  |  |  |

**** Although pregnancy is not a disease, the parturient’ s physiologic state is significantly altered from when the woman is not pregnant, hence the assignment of ASA 2 for a woman with uncomplicated pregnancy.
**The addition of “E” denotes Emergency surgery: (An emergency is defined as existing when delay in treatment of the patient would lead to a significant increase in the threat to life or body part)***

***Reference: America Society of Anesthesiologist 2025 [Accessed Feb 10, 2025: https://www.asahq.org/standards-and-practice-parameters/statement-on-asa-physical-status-classification-system]***

# Definitions of anaesthetic complications and Surgical complications

**Definitions of anaesthetic complications**

The following definitions are provided for guidance where the nature of a possible complication following anaesthesia is uncertain.

- **Failed intubation:**  Failure to place the endotracheal tube after multiple intubation attempts.
- **Aspiration**: Regurgitation or vomiting of gastric contents which has passed through the larynx into the trachea or tracheobronchial tree.
- **Cardiac arrest:** Cardiac arrest associated with the induction or maintenance of general anaesthesia, regional anaesthesia or airway manipulation. Cardiac arrest is defined as the cessation of cardiac mechanical activity, as confirmed by the absence of signs of circulation. ECG changes may corroborate the incidence of cardiac arrest.
- **Severe hypoxia:** Hypoxia with a peripheral saturation of <90% on pulse oximetry, or clinical impression of hypoxia in the absence of a pulse oximeter.

Definitions and grading of surgical complications

The following definitions and grading are provided for guidance where the nature and
severity of a possible complication following surgery is uncertain. These definitions are
based on the ‘Standards for definitions and use of outcome measures for clinical
effectiveness research in perioperative medicine: European Perioperative Clinical
Outcome (EPCO) definitions: a statement from the ESA-ESICM joint taskforce on
perioperative outcome measures

Acute Kidney Injury (AKI)
Acute Kidney Injury (AKI) Stage

- Mild Increase of cr=1.5-1.9 times baseline value within 7 days or ≥0.3mg/dL (27 μmol/L) within 48 hours or UOP ≤0.5 ml/kg/h for 6-12 hours/
- Moderate Increase of 2.0-2.9 times baseline value within 7 days OR UOP ≤0.5 ml/kg/h for 12 hours
- Severe Increase of 3.0 times baseline within 7 days or increase in serum creatinine to ≥4.0 mg/Dl (≥354 μmol/L) with an acute rise of >0.5 mg/Dl (>44 μmol/L) or initiation
  of renal replacement therapy OR UOP ≤0.3 ml/kg/h for 24 hours
  or Anuria for 12 hours

Acute Respiratory Distress Syndrome (ARDS)

- Respiratory failure, or new or worsening respiratory symptoms, commencing within
  one week of surgery; and a chest radiograph or computed tomography scan which
  demonstrates bilateral opacities not fully explained by effusions, lobar/lung collapse, or
  nodules; and respiratory failure not fully explained by cardiac failure or fluid overload.
  Need objective assessment (e.g. echocardiography) to exclude hydrostatic oedema if no
  risk factor is present.

Severity grading:

- Mild: PaO2:FiO2 between 200 and 300 mmHg with PEEP or CPAP ≥5 cmH2O
- Moderate: PaO2:FiO2 between 100 and 200 mmHg with PEEP ≥5 cmH2O
  Severe: PaO2:FiO2 ≤100 mmHg with PEEP ≥5 cmH2O
  **Guidance:**If altitude is higher than 1000 m, a correction factor should be calculated as follows:
  (PaO2:FiO2 x barometric pressure/760 mmHg. PEEP, positive end-expiratory
  pressure; CPAP, non-invasive continuous positive airways pressure

**Anastomotic breakdown**
Leak of luminal contents from a surgical connection between two hollow viscera. The
luminal contents may emerge either through the wound or at the drain site, or they may
collect near the anastomosis, causing fever, abscess, septicaemia, metabolic disturbance
and/or multiple-organ failure. The escape of luminal contents from the site of the
anastomosis into an adjacent localised area, detected by imaging, in the absence of
clinical symptoms and signs should be recorded as a sub-clinical leak.
Severity grading:

- Mild: Results in only temporary harm and would not usually require specific clinical
  treatment.
- Moderate: More serious complication but one which does not usually result in
  permanent harm or functional limitation. Usually requires clinical treatment.
- Severe: Results in significant prolongation of hospital stay and/or permanent functional
  limitation or death. Almost always requires clinical treatment

**Arrhythmia**Electrocardiograph (ECG) evidence of cardiac rhythm disturbance.
Severity grading:

- Mild: Results in only temporary harm and would not usually require specific clinical
  treatment.
- Moderate: More serious complication but one which does not usually result in
  permanent harm or functional limitation. Usually requires clinical treatment.
- Severe: Results in significant prolongation of hospital stay and/or permanent functional
  limitation or death. Almost always requires clinical treatment.

Cardiac arrest
The cessation of cardiac mechanical activity, as confirmed by the absence of signs of
circulation. ECG changes may corroborate the incidence of cardiac arrest.
Severity grading: None

(Cardiogenic) pulmonary oedema: Evidence of fluid accumulation in the alveoli due to poor cardiac function.
Severity grading:

- Mild: Results in only temporary harm and would not usually require specific clinical
  treatment.
- Moderate: More serious complication but one which does not usually result in
  permanent harm or functional limitation. Usually requires clinical treatment.
- Severe: Results in significant prolongation of hospital stay and/or permanent functional
  limitation or death. Almost always requires clinical treatment

Gastro-intestinal bleed
Unambiguous clinical or endoscopic evidence of blood in the gastro-intestinal tract. Upper gastrointestinal bleeding is that originating from the oesophagus, stomach and duodenum. Lower gastro-intestinal bleeding originates from the small bowel and colon.
Severity:

- Mild: Results in only temporary harm and would not usually require specific clinical treatment.
- Moderate: More serious complication but one which does not usually result in permanent harm or functional limitation. Usually requires clinical treatment.
- Severe: Results in significant prolongation of hospital stay and/or permanent functional limitation or death. Almost always requires clinical treatment.

Bloodstream infection
An infection which is not related to infection at another site and which meets at least
one of the following criteria:

- Patient has a recognised pathogen cultured from blood cultures which is not related to an infection at another site
- Patient has at least one of the following signs or symptoms: fever (>38°C), chills, or hypotension and at least one of the following:

1. common skin contaminant cultured from two or more blood cultures
   drawn on separate occasions
2. common skin contaminant cultured from at least one blood culture from a patient with an intravascular line, and a physician starts antimicrobial
   therapy
3. positive blood antigen test

Severity:

- Mild: Results in only temporary harm and would not usually require specific clinical treatment.
- Moderate: More serious complication but one which does not usually result in permanent harm or functional limitation. Usually requires clinical treatment.
- Severe: Results in significant prolongation of hospital stay and/or permanent functional limitation or death. Almost always requires clinical treatment.

Myocardial infarction
Increase in serum cardiac biomarker values (preferably cardiac troponin) with at least
one value above the 99th percentile upper reference limit and at least one of the
following criteria:
1. Symptoms of ischemia
2. New or presumed new ST-segment or T-wave ECG changes or new left bundle branch block
3. Development of pathological Q-waves on ECG
4. Radiological or echocardiographic evidence of new loss of viable myocardium or new regional wall motion abnormality
5. Identification of an intra-coronary thrombus at angiography or autopsy

Severity grading:

- Mild: Results in only temporary harm and would not usually require specific clinical treatment.
- Moderate: More serious complication but one which does not usually result in permanent harm or functional limitation. Usually requires clinical treatment.
- Severe: Results in significant prolongation of hospital stay and/or permanent functional limitation or death. Almost always requires clinical treatment.

Pneumonia
Chest radiographs with new or progressive and persistent infiltrates, or consolidation,
or cavitation, and at least one of the following:
1. fever (>38°C) with no other recognized cause
2. leucopaenia (<4,000 white blood cells/mm3) or leucocytosis (>12,000 white
blood cells/mm3)
3. for adults >70 years old, altered mental status with no other recognised cause;
and at least two of the following:
1. new onset of purulent sputum or change in character of sputum, or increased
respiratory secretions, or increased suctioning requirements
2. new onset or worsening cough, or dyspnoea, or tachypnoea
3. rales or bronchial breath sounds
4. worsening gas exchange (hypoxaemia, increased oxygen requirement or
increased ventilator demand)

Guidance: Two radiographs are required for patients with underlying pulmonary or cardiac disease. The definition may be used to identify ventilator associated pneumonia.
Severity:

- Mild: Results in only temporary harm and would not usually require specific clinical treatment.
- Moderate: More serious complication but one which does not usually result in permanent harm or functional limitation. Usually requires clinical treatment.
- Severe: Results in significant prolongation of hospital stay and/or permanent functional limitation or death. Almost always requires clinical treatment

Postoperative haemorrhage
Blood loss occurring within 72 hours after the end of surgery which would normally result in transfusion of blood. Gastro-intestinal bleeding is defined above.
Severity:

- Mild: Not applicable
- Moderate: More serious complication but one which does not usually result in permanent harm or functional limitation. Usually requires clinical treatment.
- Severe: Results in significant prolongation of hospital stay and/or permanent functional limitation or death. Almost always requires clinical treatment.

Pulmonary embolism (PE)
A new blood clot or thrombus within the pulmonary arterial system.
Guidance: Appropriate diagnostic tests include scintigraphy and CT angiography. Plasma D-dimer measurement is not recommended as a diagnostic test in the first three weeks following surgery.
Severity:

- Mild: Results in only temporary harm and would not usually require specific clinical treatment.
- Moderate: More serious complication but one which does not usually result in permanent harm or functional limitation. Usually requires clinical treatment.
- Severe: Results in significant prolongation of hospital stay and/or permanent functional limitation or death. Almost always requires clinical treatment

Stroke
Embolic, thrombotic, or haemorrhagic cerebral event with persistent residual motor, sensory, or cognitive dysfunction (e.g. hemiplegia, hemiparesis, aphasia, sensory deficit, impaired memory).
Severity:
Mild: Results in only temporary harm and would not usually require specific clinical treatment.
Moderate: More serious complication but one which does not usually result in permanent harm or functional limitation. Usually requires clinical treatment.
Severe: Results in significant prolongation of hospital stay and/or permanent functional limitation or death. Almost always requires clinical treatment

Surgical site infection (superficial)
Infection involving only superficial surgical incision which meets the following criteria:
1. Infection occurs within 30 days after surgery and
2. Involves only skin and subcutaneous tissues of the incision and
3. The patient has at least one of the following:
a. purulent drainage from the superficial incision
b. organisms isolated from an aseptically obtained culture of fluid or tissue
from the superficial incision and at least one of the following signs or
symptoms of infection: pain or tenderness, localized swelling, redness, or
heat, or superficial incision is deliberately opened by surgeon and is
culture positive or not cultured. A culture-negative finding does not meet
this criterion.
c. diagnosis of a incisional surgical site infection by a surgeon or attending
physician
Severity:
Mild: Results in only temporary harm and would not usually require specific clinical treatment.
Moderate: More serious complication but one which does not usually result in permanent harm or functional limitation. Usually requires clinical treatment.
Severe: Results in significant prolongation of hospital stay and/or permanent functional limitation or death. Almost always requires clinical treatment

Surgical site infection (deep)
An infection which involves both superficial and deep parts of surgical incision and
meets the following criteria:
1. Infection occurs within 30 days after surgery if no surgical implant is left in place or one year if an implant is in place and
2. The infection appears to be related to the surgical procedure and involves deep
soft tissues of the incision (e.g. fascial and muscle layers) and
3. The patient has at least one of the following:
a. purulent drainage from the deep incision but not from the organ/space
component of the surgical site
b. a deep incision spontaneously dehisces or is deliberately opened by a
surgeon and is culture-positive or no cultures were taken whilst the
patient has at least one of the following signs or symptoms of infection:
fever (>38°C) or localized pain or tenderness. A culture-negative finding
does not meet this criterion.
c. an abscess or other evidence of infection involving the deep incision is
found on direct examination, during surgery, or by histopathologic or
radiologic examination
d. diagnosis of a deep incisional surgical site infection by a surgeon or
attending physician
Severity:
Mild: Results in only temporary harm and would not usually require specific clinical treatment.
Moderate: More serious complication but one which does not usually result in permanent harm or functional limitation. Usually requires clinical treatment.
Severe: Results in significant prolongation of hospital stay and/or permanent functional limitation or death. Almost always requires clinical treatment

Surgical site infection (organ/space)
An infection which involves any part of the body excluding the fascia or muscle layers
and meets the following criteria:
1. Infection occurs within 30 days after surgery and
2. The infection appears to be related to the surgical procedure and involves any
part of the body, excluding the skin incision, fascia, or muscle layers, that is
opened or manipulated during the operative procedure and
3. The patient has at least one of the following:
a. purulent drainage from a drain that is placed through a stab wound into
the organ/space
b. organisms isolated from an aseptically obtained culture of fluid or tissue
in the organ/ space
c. an abscess or other evidence of infection involving the organ/space that is
found on direct examination, during reoperation, or by histopathologic or
radiologic examination
d. diagnosis of an organ/space surgical site infection by a surgeon or
attending physician
Severity:
Mild: Results in only temporary harm and would not usually require specific clinical
treatment.
Moderate: More serious complication but one which does not usually result in permanent harm or functional limitation. Usually requires clinical treatment.
Severe: Results in significant prolongation of hospital stay and/or permanent functional limitation or death. Almost always requires clinical treatment

Urinary tract infection
An infection associated with at least one of the following signs or symptoms which should be identified within a 24 hour period; fever (>38 °C), urgency, frequency, dysuria, suprapubic tenderness, costovertebral angle pain or tenderness with no other recognised cause, and a positive urine culture of ≥105 colony forming units/mL with no more than two species of microorganisms.
Severity:
Mild: Results in only temporary harm and would not usually require specific clinical treatment.
Moderate: More serious complication but one which does not usually result in permanent harm or functional limitation. Usually requires clinical treatment.
Severe: Results in significant prolongation of hospital stay and/or permanent functional limitation or death. Almost always requires clinical treatment.

## Appendix: Result


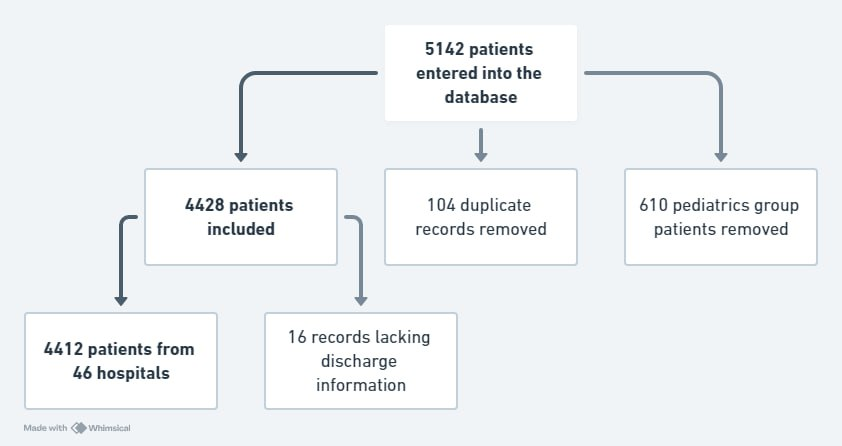


**Figure S1**: Ethiopia surgical outcome and patient recruitment

Table S1: Comorbidity in Ethiopian Surgical outcome study (EthioSOS)

| **Comorbidity** | **All patients**  **(n=4412)** | **Patients with complications (n=873)** | **Patients with no complications (n=3538)** | **Patients who died (n=17)** | **Patients who survived (n=4395)** |
| --- | --- | --- | --- | --- | --- |
| CHF | 13(0.3%) | 6(0.7%) | 7(0.2%) | 0(0%) | 13(0.3%) |
| PVD | 10 (0.2%) | 4(0.5%) | 6(0.2%) | 0(0%) | 10(0.2%) |
| Hemiplegia | 5 (0.1%) | 2(0.2%) | 3(0.1%) | 0(0%) | 5(0.1%) |
| IHD | 7 (0.2%) | 5(0.6%) | 2(0.1%) | 1(5.9%) | 6(0.1%) |
| HIV/AIDS | 47(1.1%) | 11(1.3%) | 36(1.0%) | 1(5.9%) | 46(1.0%) |
| Hypertension | 243(5.5%) | 82(9.4%) | 160(4.5%) | 1(5.9%) | 242(5.5%) |
| Stroke/TIA | 4(0.1%) | 2(0.2%) | 2(0.1%) | 0(0%) | 4(0.1%) |
| PUD | 15(0.3%) | 9(1.0%) | 6(0.2%) | 0(0%) | 15(0.3%) |
| Connective tissue disease | 2 (0.05%) | 0.(0%) | 2(0.06%) | 0(0%) | 2(0.05%) |
| CKD (moderate to severe) | 11(0.25%) | 5(0.6%) | 6(0.2%) | 0(0%) | 12(0.3%) |
| Diabetes Mellitus | 106(2.4%) | 37(4.2%) | 69(1.9%) | 1(5.9%) | 105(2.4%) |
| Liver disease | 6(0.14%) | 2(0.2%) | 4(0.1%) | 0(0%) | 6(0.13%) |
| Solid tumor | 48(1.1%) | 12(1.4%) | 36(1.0%) | 2(11.8%) | 46(1.0%) |
| Leukemia | 1(0.02%) | 0(0%) | 1(0.03%) | 0(0%) | 1(0.02%) |
| COPD/Asthma | 34(0.8%) | 13(1.5%) | 21(0.6%) | 1(5.9%) | 33(0.75%) |
| None | 3764(85.3%) | 623(71.4%) | 3141(88.8%) | 11(64.7%) | 3753(85.4%) |

Table S2: Association between primary indication for surgery and postoperative complications and in-hospital mortality.

| **Indication of surgery** | **All patients**  **(n=4412)** | **Complication n = 873** | **No complication n =**3538 | **OR (95%CI)** | **p-value** | **Died**  **(n=17)** | **Survived**  **(n=4394)** | **OR (95%CI)** | **p-value** |
| --- | --- | --- | --- | --- | --- | --- | --- | --- | --- |
| Non-communicable | 1724(39.1%) | 387 (44.3%) | 1,337(37.8%) | Ref |  | 10(58.9%) | 1714(39.0%) | Ref | NA |
| Cesarean section | 1639(37.2%) | 211(24.2%) | 1,428(40.4%) | 0.51(0.42, 0.61) | **<0.001*** | 2(11.8%) | 1637(37.2%) | 0.21(0.05, 0.96) | **0.044*** |
| Infection | 388(8.8%) | 114(13.1%) | 274(7.7%) | 1.44(1.12,1.84) | **0.004*** | 2(11.8%) | 386(8.8%) | 0.89(0.20, 4.07) | 0.879 |
| Trauma | 660(15.0%) | 161(18.4%) | 499(14.1%) | 1.11(0.90, 1.37) | 0.312 | 3(17.6%) | 657(14.9%) | 0.78(0.21, 2.85) | 0.710 |

Data presented as n/N (%); *: shows significant difference at P<0.05; Ref: Reference category; CI: Confidence interval; OR: Odds ratio

Table S3: Comparison between patients in ISOS, ASOS, NiSOS, SASOS, and Ethio-SOS

| **Variable** | **ISOS** | **ASOS (Elective)** | **NiSOS** | **SASOS** | **Ethio-SOS** |
| --- | --- | --- | --- | --- | --- |
|  | **n = 44814** | **n = 4874** | **n = 1425** | **n=** **3927** | **n = 4412** |
| Age, (Mean) SD | 55.3(17.1) | 38.5(16.1) | 35.4(21.5) | 43.5 (17.6) | 35.1(14.7) |
| ASA I (n%) | 11227(25.1) | 1737 (44.9) | 604(42.4) | 1743 (44.7) | 2113 (47.9) |
| Minor surgery(n%) | 8411(18.8) | 1140 (29.5) | 161(11.3) | 1403 (36.1) | 303(6.9) |
| Mortality(n%) | 207(0.5) | 48(1.0) | 22(1.5) | 123 (3.1%) | 17(0.4) |
| Complications(n%) | 7508(16.8) | 624(13.4) | 264(18.5) | NA | 873 (19.8) |
| Mortality after complications(n%) | 207(2.8) | 30(4.8) | 16(6.0) | NA | 13(1.5%) |

Data presented as n/N (%); ASOS: African surgical outcome study; ISOS: International surgical outcome study; Nigerian surgical outcome study; Ethio-SOS; Ethiopian surgical outcome study; SASOS: South Africa surgical outcome study; NA: Not applicable.

Table S4*:* Incidence of mortality associated with Clavien-Dindo classification of surgical complications

| **S. N** | **Clavien-Dindo classification** | **Numbers of patients (n)** | **Mortality rate (%)** |
| --- | --- | --- | --- |
| 1 | Grade I | 282 | 0/282(0%) |
| 2 | Grade II | 406 | 2/406(0.5%) |
| 3 | Grade IIIA | 86 | 0/86(0%) |
| 4 | Grade IIIB | 63 | 3/63(4.8%) |
| 5 | Grade IV A | 28 | 5/28(18.5%) |
| 6 | Grade IV B | 8 | 3/8(37.5%) |
|  | **Total** | **873** | **13/873(1.5%)** |

Table S5: *Multivariable logistic regression for adjusted predictors of postoperative complication.*

| **Variables** | | **AOR (95%CI)** | **P-value** |
| --- | --- | --- | --- |
|  |  |  |  |
| Age category | 18-29 years |  | |
|  | 30-69 years | 1.03(0.86,1.23) | 0.717 |
|  | ≥ 70 years | 1.40(0.96,2.03) | 0.077 |
| Sex | Female |  | |
|  | Male | 0.85(0.69,1.05) | 0.126 |
| Current smoker | No |  | |
|  | Yes | 2.47(1.60,3.80) | **<0.001*** |
| Comorbidity | No |  | |
|  | Yes | 2.63(2.15,3.23) | <**0.001*** |
| Cancer surgery | No |  | |
|  | Yes | 1.60(1.17,2.19) | **0.003*** |
| Time of surgery | Elective |  | |
|  | Urgent | 1.19(0.94,1.50) | 0.142 |
|  | Emergency | 1.44(1.16,1.79) | **0.001*** |
| Indication of surgery | NCD |  | |
|  | CS | 0.80(0.53,1.21) | 0.296 |
|  | Infection | 1.29(0.97,1.72) | 0.077 |
|  | Trauma | 1.15(0.85,1.56) | 0.356 |
| Type of surgery | Minor |  | |
|  | Intermediate | 1.08(0.75, 1.56) | 0.675 |
|  | Major | 1.74(1.38,1.92) | **0.002*** |
| ASA score | I |  | |
|  | II | 0.93(0.78,1.1) | 0.427 |
|  | III | 1.09(0.74,1.60) | 0.653 |
|  | ≥ IV | 4.0(2.17,7.40) | **<0.001*** |
| Surgery type | Gynecology /Obstetrics |  | |
|  | Urology | 1.40(0.85,2.31) | 0.182 |
|  | Cardio-thoracic/Vascular | 1.30(0.70,2.42) | 0.399 |
|  | Neurology | 0.76(0.46,1.23) | 0.264 |
|  | Gastro-intestinal | 1.55(1.02,2,36) | **0.043*** |
|  | Orthopedics | 1.64(1.01,2.69) | **0.049*** |
|  | Others | 1.81(1.13,2.90) | **0.014*** |

*: significant < 0.05; ASA: American Society of Anesthesiologists; CS: cesarean section; NCD: Non-communicable disease, AOR: Adjusted Odds ratio.

Table S6: Characteristics of Patients versus in-hospital mortality

| Variables | | 7-day mortality | | Fisher exact (p-value) | OR (95%CI) |
| --- | --- | --- | --- | --- | --- |
|  |  | **Patients who died(n=17)** | **Patients who survived (n=4395)** |  |  |
| Age category | 18-29 years | 5(29.4%) | 2,080 (47.3%) | 0.034* | Ref |
|  | 30-69 years | 9(52.9) | 2,133(48.5%) |  | 1.76(0.59,5.25) |
|  | ≥ 70 years | 3(17.6%) | 182 (4.1%) |  | 6.86(1.62, 28.92) |
| Sex | Female | 8(47.1%) | 2764(62.9%) | 0.210 | Ref |
|  | Male | 9(52.9%) | 1631(37.1%) |  | 1.91(0.73,4.95) |
| Comorbidity | No | 11(64.7%) | 3753(85.4%) | 0.025* | Ref |
|  | Yes | 6(35.3%) | 622(14.2%) |  | 3.29(1.21,8.93) |
| Cancer surgery | No | 12(70.6%) | 4132(94.0%) | 0.003* | Ref |
|  | Yes | 5(29.4%) | 259(6.0%) |  | 6.65(2.32, 19.01) |
| Time of surgery | Elective | 3(17.6%) | 1360(31.0%) | 0.198 | Ref |
|  | Urgent | 2(11.8%) | 969(22.1%) |  | 0.94(0.16, 5.61) |
|  | Emergency | 12(70.6%) | 2061(46.9%) |  | 2.64(0.74,9.37) |
| Indication of surgery | NCD | 10(58.9%) | 1714(39.0%) | 0.103 | Ref |
|  | CS | 2(11.8%) | 1637(37.2%) |  | 0.21(0.05, 0.96) |
|  | Infection | 2(11.8%) | 386(8.8%) |  | 0.89(0.20, 4.07) |
|  | Trauma | 3(17.6%) | 657(14.9%) |  | 0.78(0.21, 2.85) |
| Type of surgery | Minor | 0(0%) | 303 (6.9%) | 00.005* | Ref |
|  | Intermediate | 1(5.9%) | 1,637 (37.3%) |  | NA |
|  | Major | 16(94.1%) | 2454(55.8%) |  | 10.67(1.41,80.56) |
| ASA score | I | 5(29.4%) | 2108(48.0%) | <0.001* | Ref |
|  | II | 5(29.4%) | 2069(47.1%) |  | 1.02(0.29,3.52) |
|  | III | 5(29.4%) | 171 (3.9%) |  | 12.33(3.53, 43.0) |
|  | ≥ IV | 2(11.8%) | 47(1.1%) |  | 17.94(3.40, 94.84) |
| Surgery type | Gynecology /Obstetrics | 3(17.65%) | 1859(42.32%) | 0.004* | Ref |
|  | Urology | 1(5.88%) | 289(6.58%) |  | 2.14(0.22,20.68) |
|  | Cardio-thoracic/Vascular | 1(5.88%) | 102(2.32%) |  | 6.07(0.62,58.91) |
|  | Neurology | 0(0%) | 344(7.83%) |  | NA |
|  | Gastro-intestinal | 11(64.71%) | 924(21.03%) |  | 7.37(2.05,26.50) |
|  | Orthopedics | 1(5.88%) | 518(11.79%) |  | 1.20(0.12,11.52) |
|  | Others | 0(0%) | 357(8.13%) |  | NA |

All data presented as n/N (%); ASA: American Society of Anesthesiologists; CS: cesarean section; NA: Not applicable; NCD: Non-communicable disease, OR: Odds ratio.

Table S7*: Region-specific reported mortality, postoperative complications, and critical care admission*

| **Region** | **Number of Patients (n=4412)** | **In-hospital mortality n/N (%)** | **Post-op complications**  **n/N (%)** | **Critical care admissions n/N (%)** | **Duration of Hospital stay Mean (SD)** |
| --- | --- | --- | --- | --- | --- |
| Addis Abeba | 902 | 4/902(0.4) | 189/902(20.9) | 51/902(5.7) | 3.5(1.6) |
| Oromia | 724 | 2/724(0.3) | 130/724(18.0) | 10/720(1.4) | 3.5(1.5) |
| Amhara | 764 | 5/764(0.7) | 129/764(16.9) | 16/760(2.1) | 3.7(1.4) |
| South-west Ethiopia | 62 | 2/62(3.2) | 16/62(25.8) | 5/62(8.1) | 4.3(1.8) |
| Central Ethiopia | 344 | 0/344(0) | 32/344(9.3) | 4/344(1.2) | 2.5(1.2) |
| South Ethiopia | 385 | 1/385(0.3) | 171/385(44.4) | 17/385(4.4) | 4.2(1.2) |
| Tigray | 148 | 0/148(0) | 28/148(18.9) | 3/148(2.0) | 2.9(1.4) |
| Sidama | 560 | 2/560(0.4) | 103/560(18.4) | 25/559(4.5) | 3.5(1.6) |
| Benishangul-Gumuz | 190 | 0/190(0) | 5/190(2.6) | 1/189(0.5) | 3.2(1.4) |
| Afar | 17 | 0/17(0) | 2/17(11.8) | 0/17(0) | 4.0(1.8) |
| Dire-dawa | 76 | 0/76(0) | 7/76(9.3) | 4/75(5.3) | 5.3(1.8) |
| Gambella | 37 | 0/37(0) | 11/37(29.7) | 1/37(2.7) | 4.8(1.7) |
| Hareri | 54 | 1/54(1.8) | 14/54(25.9) | 11/53(20.7) | 3.3(1.4) |
| Somali | 149 | 0/149(0) | 36/149(24.2) | 5/149(3.4) | 4.1(1.6) |

Table S8*: The hospital level-specific reported mortality, postoperative complications, and critical care admission*

| **Region** | **Number of Patients (n=4412)** | **In-hospital mortality**  **n/N (%)** | **Post-op complications**  **n/N (%)** | **Critical care admissions n/N (%)** | **Duration of Hospital stay Mean (SD)** |
| --- | --- | --- | --- | --- | --- |
| Primary hospital | 39 | 0/39(0) | 5/39(12.8) | 2/39(5.1) | 5.2(1.5) |
| General hospital | 1739 | 5/1739(0.3) | 387/1739(22.2) | 48/1734(2.8) | 3.8(1.5) |
| Specialized hospitals | 2634 | 12/2634(0.5) | 481/2634(18.3) | 103/2627(3.9) | 3.4(1.6) |
