# Supplementary material for: The Ethiopian Surgical Outcome Study (Ethio-SOS): a 7-day multicentre national prospective observational cohort study
Source: BMJ Glob Health. 2025 Sep 29;10(9):e020147. doi: 10.1136/bmjgh-2025-020147 (PMC12481335; doi:10.1136/bmjgh-2025-020147)
Supplement: Supplementary file 3 [file bmjgh-10-9-s003.docx]

# Appendix 3: Authorship appendix to “The Ethiopian Surgical Outcome Study (Ethio-SoS): a multicenter national prospective study ‘’

#

This appendix provides further authorship detail for “**The Ethiopian Surgical Outcome Study (Ethio-SoS): a multicenter national prospective study”**

Contents

[Appendix 3: Authorship appendix to “The Ethiopian Surgical Outcome Study (Ethio-SoS): a multicenter national prospective study ‘’ 1](#_Toc204629599)

[Ethiopian Surgical Outcomes Study (Ethio-SOS) investigators 2](#_Toc204629600)

[Affiliations 2](#_Toc204629601)

[Guarantor Author 3](#_Toc204629602)

[Authors’ Contributions 3](#_Toc204629603)

[Data collection or critical feedback on data collection tools 3](#_Toc204629604)

[Developing methods and hospital leaders 4](#_Toc204629605)

[Providing critical feedback on methods or results 4](#_Toc204629606)

[Drafting the work or revising it critically for important intellectual content 4](#_Toc204629607)

[Accessed and verified Data 5](#_Toc204629608)

### Ethiopian Surgical Outcomes Study (Ethio-SOS) investigators

Atalel Fentahun Awedew, Fitsum Kifle Belachew, Katherine R. Iverson, Tesfay Yohannes, Kokeb Desita Belihu, Abiy Dawit Tantu, Leake Gebrargs Gebreslase, Masresha Gebru Teklehaimanot, Kalkidan Kifle, Nigat Amsalu Addis, Peniel Kenna Dula, Prof Bruce Biccard ,Prof. Andualem Deneke, Bezaye Zemed, Abdi Beshir Mohammed, Abdilhey Hassen Bushra, Abdinasir Mohamed, Abdureuf Misganew Adane, Abiy Dawit Tantu, Ahmed Abdella, Alazar Berhe Aregawi, Andualem Dagne Tebkew, Aregawi Tewelde Teklu, Asefa Chukala Hawas, Ayele Bekele Weyesa, Bedemariam Tadesse, Bekele Abera Mekonen, Beshea Mitiku Fufa, Bethelhem Yaynemsa Sequr, Binyam Yohannes, Birhanu Ayana Chekol, Bizuneh Sime Debela, Ermiyas Belay, Iman Zeidan, Kidist Hunegn Setargew, Mejudin Kedir Abdella, Mengistu Ayele Yigzaw, Mestet Yibeltal Shiferaw, Mohammed Seid, Nebiyou Bayleyegn, Nina Berr, Rafika Amin Omer ,Samuel Zerihun, Segni Melese Alemu, Selam Daniel Meshesha, Tadese Fenta Gela, Teame Tekleab Gebremedhin, Teka Kemal Abagojam, Tesfagenet Anamo, Tsion Wolanewos Asfaw, Yelfgn Amare Fikadu, Yiacob Tilahun Kibret, Yihdego Bitsa Gebrezgi, Yohanes Yoseph Mesfine, Yonatan Abie Tsegaye, Yordanos Teshager Eshete, Zaid Hadgu Tesfay, Zekaryas Belete Tilahun, Siraj Ahmed Ali

# Affiliations

Department of Surgery (A F Awedew MD), Department of internal medicine (T W Asfaw MD, K H Setargew MD), Debre Tabor University, Debre Tabor, Ethiopia; Global Partner for Improving Surgical System (P K Dula MSc, K Kifle BSc, T Yohannes MSc), Network for Perioperative and Critical Care (GPISS-N4PCc), Addis Ababa, Ethiopia; Department of Anesthesiology (F K Alebachew PhD,K D Belihu MSc, B Zemed MSc), Debre Birhan University, Debre Birhan, Ethiopia; Department of Gynecology and Obstetrics (N A Addis MD), University of Gondar, Gondar, Ethiopia; Department of Anaesthesia and Perioperative Medicine (Prof B Biccard PhD), University of Cape Town, Cape Town, South Africa; Department of Surgery (K R. Iverson MD), Medical College of Wisconsin/ Froedtert Hospital, USA; Department of surgery (Prof A Deneke MD), Department of Gynecology and Obstetrics (A Abdella MD), Addis Ababa University, Addis Ababa, Ethiopia; Department of surgery (N Bayleyegn MD), Jimma University, Jimma, Ethiopia; Department of Surgery (S M Alemu MD), Dembi Dolo University, Dembi Dolo, Ethiopia; Department of Urology (M K Abdella MD), Werabe University, Werabe, Ethiopia; Department of Surgery (A B Aregawi MD), Department of Neurosurgery (M A Yigzaw MD), Hawassa University, Hawassa, Ethiopia; Department of Surgery (B Yohannes MD), Saint Paul Millenium Medical college, Addis Ababa, Ethiopia; Department of Surgery (B S Debela MD), Yirgalem Medical College, Yirgalem, Ethiopia; Department of Orthopedics and Trauma Surgery (Y A Tsegaye MD), Lahey Hospital*and Debre Tabor University, Ethiopia; Department of Surgery (A D Tebkew MD, B Tadesse MD), Bahir Dar University, Bahir Dar, Ethiopia; ImPACT Africa expert data manager in Department of Anesthesiology (M G Teklehaimanot MSc), Mekelle University, Mekelle, Ethiopia; Department of Surgery (B A Mekonen MD), Bule Hora University, Guji, Ethiopia; Department of Orthopedics and Trauma Surgery (A C Hawas MD), Adama Medical College, Adama, Ethiopia; Department of Neurosurgery (M Y Shiferaw MD), Injibara University, Injibara Ethiopia; Department of Anesthesia (L G Gebreslase MSc), Aksum university, Aksum, Ethiopia; Department of internal medicine (T Anamo MD), Wolaita Sodo university, Wolaita, Ethiopia; Department of Surgery (B A Chekol MD), Debre Markos University, Debre Markos, Ethiopia; Department of Surgery (S Zerihun MD), Minilik II Comprehensive Hospital, Addis Ababa, Ethiopia; Department of Surgery (A M. Adane MD), Wollo University, Dessie, Ethiopia; Department of Surgery (Y Y Mesfine MD), Department of Gynecology and Obstetrics (B Y Sequr MD), Mizan Aman University, Mizan Aman, Ethiopia; Department of Surgery (A B Mohammed MD), Department of Biostatistics (E Belay MSc), Department of Gynecology and Obstetrics (A H Bushra MD), Welkite University, Welkite, Ethiopia; Department of Surgery (Z B Tilahun MD), Yikatit 12 Medical College, Addis Ababa, Ethiopia; Department of Surgery (A Mohamed MD), Jigjiga University, Jigjiga , Ethiopia; Department of Surgery (T F Gela MD), Debark General Hospital, Debark, Ethiopia; Department of Anesthesiology (S D Meshesha MD), Saint Petros Specialized Hospital, Addis Ababa , Ethiopia; Department of Surgery (Y T Kibret MD), Sabian General hospital, Dire Dawa, Ethiopia; Department of Surgery (M Seid MD), Dire Dawa University, Dire Dawa, Ethiopia; Department of Surgery (Y A Fikadu MD), ALERT specialized Hospital, Addis Ababa, Ethiopia; Department of Surgery (B M Fufa MD), Wollega University, Nekemte, Ethiopia; Department of Surgery (Y T Eshete MD), Gandi Memorial Hospital, Addis Ababa, Ethiopia; Medical Service (A D Tantu PhD), Minister of Health, Addis Ababa, Ethiopia; Department of Surgery (N Berr MD), Zewuditu memorial Hospital, Addis Ababa, Ethiopia; Department of Anesthesia (A B Weyesa MSc), Arsi University, Asela, Ethiopia; Department of Nursing (R A Omer, BSc), Jugol general hospital, Harra, Ethiopia; Medical Service Directorate (T K Abagojam MPH), Gambella Regional Beuro and Gambella General hospital, Gambella, Ethiopia; Medical Service Directorate (I Zeidan, MD), Harari regional health bureau, Harrar, Ethiopia; Department of Anesthesia (Z H Tesfay BSc), Awash Referral Hospital, Mekelle, Ethiopia; Department of Anesthesia (Y B Gebrezgi MSc), Adigrat General Hospital, Adigrat, Ethiopia; Department of Anesthesiology (T T Gebremedhin MSc), Mekelle University, Mekelle, Ethiopia Department of Anesthesia (A T Teklu MSc), Mekelle general hospital, Mekelle, Ethiopia ; Department of Anesthesiology (S A Ali), Dilla University, Dilla, Ethiopia

## Guarantor Author

Atalel Fentahun Awedew is responsible for the overall content as guarantor

## Authors’ Contributions

### Data collection or critical feedback on data collection tools

Atalel Fentahun Awedew, Fitsum Kifle Belachew, Katherine R. Iverson, Tesfay Yohannes, Kokeb Desta, Kalkidan Kifle, Prof. Andualem Deneke, Nigat Amsalu Addis, Prof Bruce Biccard, Masresha Gebru Teklehaimanot, Leake Gebrargs Gebreslase, Peniel Kenna Dula, Bezaye Zemed, Abdi Beshir Mohammed, Abdilhey Hassen Bushra, Abdinasir Mohamed, Abdureuf Misganew Adane, Abiy Dawit Tantu, Ahmed Abdella, Alazar Berhe Aregawi, Andualem Dagne Tebkew, Aregawi Tewelde Teklu, Asefa Chukala Hawas, Ayele Bekele Weyesa, Bedemariam Tadesse, Bekele Abera Mekonen, Beshea Mitiku Fufa, Bethelhem Yaynemsa Sequr, Binyam Yohannes, Birhanu Ayana Chekol, Bizuneh Sime Debela, Ermiyas Belay, Elubabor Buno^,^ Iman Zeidan, Kidist Hunegn Setargew, Mejudin Kedir Abdella, Mengistu Ayele Yigzaw, Mestet Yibeltal Shiferaw, Mohammed Seid, Nebiyou Bayleyegn, Nina Berr, Rafika Amin Omer ,Samuel Zerihun, Segni Melese Alemu, Selam Daniel Meshesha, Tadese Fenta Gela, Teame Tekleab Gebremedhin, Teka Kemal Abagojam, Tesfagenet Anamo, Tsion Wolanewos Asfaw, Yelfgn Amare Fikadu, Yiacob Tilahun Kibret, Yihdego Bitsa Gebrezgi, Yohanes Yoseph Mesfine, Yonatan Abie Tsegaye, Yordanos Teshager Eshete, Zaid Hadgu Tesfay, Zekaryas Belete Tilahun

### Developing methods and hospital leaders

Atalel Fentahun Awedew, Fitsum Kifle Belachew, Katherine R. Iverson, Tesfay Yohannes, Kokeb Desta, Kalkidan Kifle, Prof. Andualem Deneke, Nigat Amsalu Addis, Prof Bruce Biccard, Masresha Gebru Teklehaimanot, Leake Gebrargs Gebreslase, Peniel Kenna Dula, Bezaye Zemed,

### Providing critical feedback on methods or results

Atalel Fentahun Awedew, Fitsum Kifle Belachew, Katherine R. Iverson, Tesfay Yohannes, Kokeb Desta, Kalkidan Kifle, Prof. Andualem Deneke, Nigat Amsalu Addis, Prof Bruce Biccard, Masresha Gebru Teklehaimanot, Leake Gebrargs Gebreslase, Peniel Kenna Dula, Bezaye Zemed, Abdi Beshir Mohammed, Abdilhey Hassen Bushra, Abdinasir Mohamed, Abdureuf Misganew Adane, Abiy Dawit Tantu, Ahmed Abdella, Alazar Berhe Aregawi, Andualem Dagne Tebkew, Aregawi Tewelde Teklu, Asefa Chukala Hawas, Ayele Bekele Weyesa, Bedemariam Tadesse, Bekele Abera Mekonen, Beshea Mitiku Fufa, Bethelhem Yaynemsa Sequr, Elubabor Buno,^,^ Binyam Yohannes, Birhanu Ayana Chekol, Bizuneh Sime Debela, Ermiyas Belay, Iman Zeidan, Kidist Hunegn Setargew, Mejudin Kedir Abdella, Mengistu Ayele Yigzaw, Mestet Yibeltal Shiferaw, Mohammed Seid, Nebiyou Bayleyegn, Nina Berr, Rafika Amin Omer ,Samuel Zerihun, Segni Melese Alemu, Selam Daniel Meshesha, Tadese Fenta Gela, Teame Tekleab Gebremedhin, Teka Kemal Abagojam, Tesfagenet Anamo, Tsion Wolanewos Asfaw, Yelfgn Amare Fikadu, Yiacob Tilahun Kibret, Yihdego Bitsa Gebrezgi, Yohanes Yoseph Mesfine, Yonatan Abie Tsegaye, Yordanos Teshager Eshete, Zaid Hadgu Tesfay, Zekaryas Belete Tilahun

### Drafting the work or revising it critically for important intellectual content

Atalel Fentahun Awedew, Fitsum Kifle Belachew, Katherine R. Iverson, Tesfay Yohannes, Kokeb Desta, Kalkidan Kifle, Prof. Andualem Deneke, Nigat Amsalu Addis, Prof Bruce Biccard, Masresha Gebru Teklehaimanot, Leake Gebrargs Gebreslase, Peniel Kenna Dula, Bezaye Zemed

# Accessed and verified Data

Atalel Fentahun Awedew, Fitsum Kifle Alebachew, Kokeb Desta, Tesfaye, Kalkidan

Declaration

F K B and BB report support for the present manuscript from the NIHR Global Health Group on Perioperative and Critical Care (NIHR 133850). F K B reports support for the present manuscript from the Smile Train Inc. (GR-0201409). A D reports support for the present manuscript from the Ministry of Health in Ethiopia (RN 879119). All other authors declare no competing interests
